# Application of a Gastroduodenal Artery Graft for Reconstruction of the Hepatic Artery during Radical Resection of Hilar Cholangiocarcinoma

**DOI:** 10.1155/2015/934565

**Published:** 2015-08-03

**Authors:** Yurong Liang, Jing Wang, Xianjie Shi, Jiahong Dong, Wanqing Gu

**Affiliations:** Department of Hepatobiliary Surgery, Hospital & Institute of Hepatobiliary Surgery, PLA General Hospital, Beijing 100853, China

## Abstract

This paper was designed to evaluate a novel surgical procedure of using a gastroduodenal artery graft for reconstruction of the hepatic artery during radical resection of hilar cholangiocarcinoma, which is citation-free and self-contained. In this paper we retrospectively analyzed the clinical data, surgical procedure, and follow-up results in nine patients who underwent hepatic artery reconstruction using a gastroduodenal artery graft during their radical resection of hilar cholangiocarcinoma and no artery thrombosis or other surgical complications were found after operation with minimum follow-up duration of three months. We recommended that a gastroduodenal artery graft was shown to be a good choice for hepatic artery resection after radical resection of hilar cholangiocarcinoma.

## 1. Introduction

The hilar is an important pathway for liver blood supply and biliary excretion. Several types of malignant tumors often affect the hepatic artery, portal vein, and biliary branches. Surgical operations for hilar cholangiocarcinoma often emphasize the resection of the affected hepatic artery and surrounding connective tissues. More recently, several types of hepatic artery resection and reconstruction procedures have been reported. In order to simplify the procedure and improve the arterial anastomosis success rate, we performed gastroduodenal artery bridging reconstruction on nine patients who underwent resection of more than 2 cm of the hepatic artery for hilar cholangiocarcinoma. This report summarizes the procedure and clinical results providing hepatobiliary surgeons with a guide for this type of arterial reconstruction surgery.

## 2. Patients and Methods

**Figure 1:**
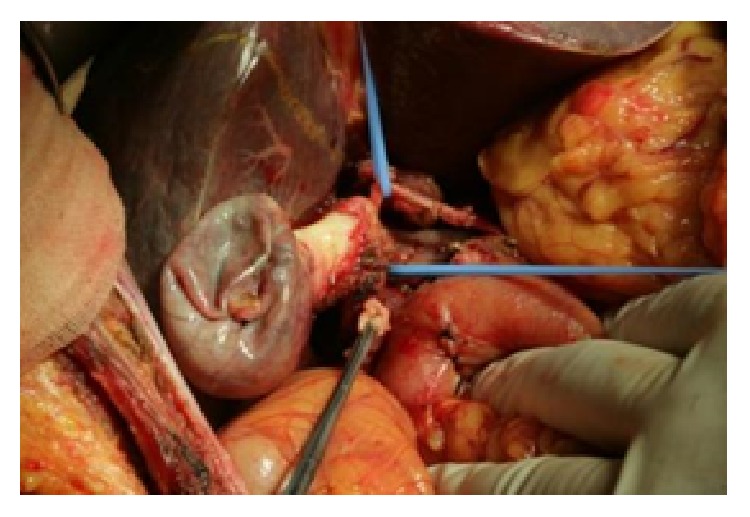
The right hepatic artery was affected. Dissect the affected hepatic artery for en bloc resection.

According to the Bismuth type [1], nine patients with hilar cholangiocarcinoma were classified into Types IIIa (2 cases), IIIb (6 cases), and IV (1 case). The patients were six males and three females, with average age of 62 years (ranging from 43 to 72 years), and their Child-Pugh scores were all Class A. In all cases, preoperative imaging studies were used to evaluate the hilar vascular involvement. Surgical exploration was also shown to be consistent with the arterial bridging standards; that is, arterial bridging would be considered for tension* in situ* reconstruction when an affected artery length was ≥2 cm. The specific steps of the procedure were undertaken. We first skeletonized the hepatoduodenal ligament as much as possible, explored the length of the artery affected by tumor, carefully dissected the hepatic artery proximal and distal to the tumor, and then resected the affected artery and tumor together. We then freed the perihepatic ligaments, cut the affected liver according to routine surgical protocols, and finally detached the affected arteries (Figure 1). For the* hepatic artery* reconstruction, we freed and dissected the gastroduodenal artery and ligated individually the encountered small branches, avoiding electrocoagulation to ensure that the adventitia of the artery was intact.

**Figure 2:**
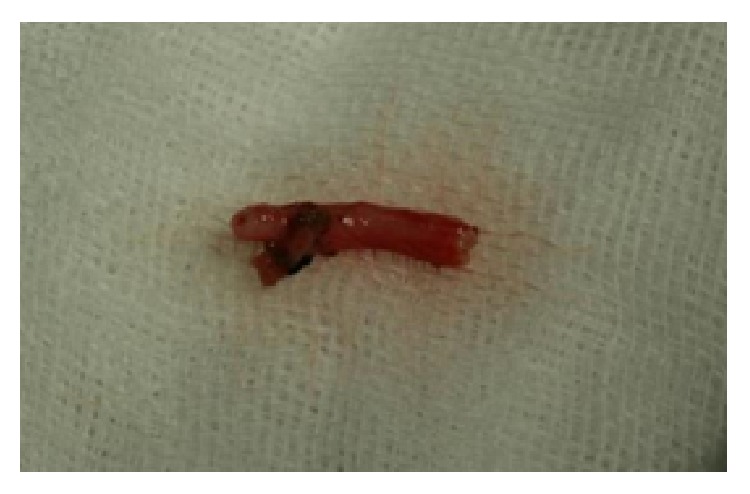
Cut off the gastroduodenal artery as a bridge for hepatic artery reconstruction.

**Figure 3:**
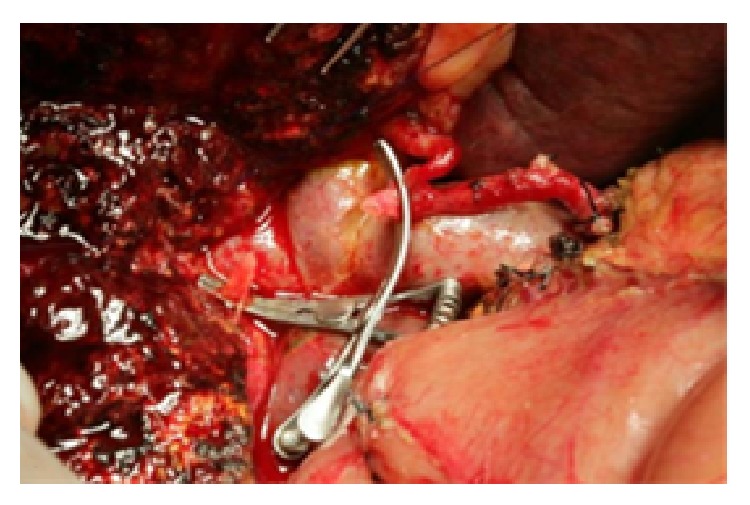
Trim the right hepatic artery proximally and distally after resection of the tumor and affected artery.

**Figure 4:**
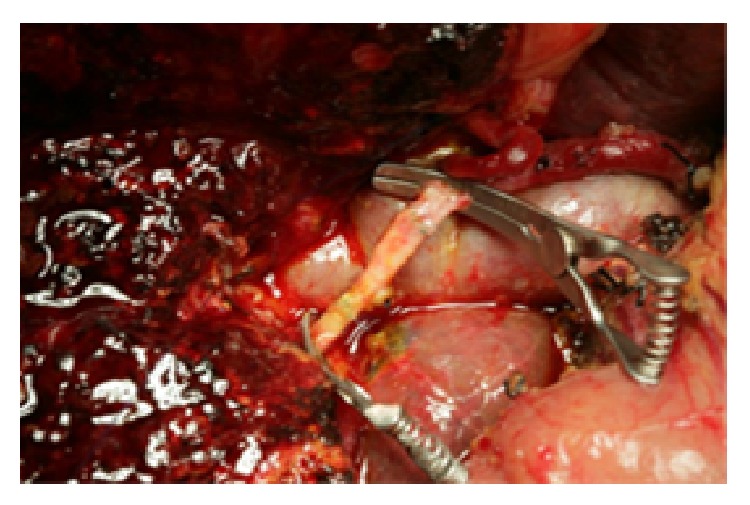
The right hepatic artery after bridging reconstruction.

For the gastroduodenal artery, we, respectively, blocked and cut from the beginning and distal end of the hepatic arteries to the upper edge of the pancreas using a sharp blade and then sutured the ends with a 6-0 Prolene. We placed the cut arterial segments into 4°C heparin (10%) in saline, for backup use after trimming (Figure 2). We trimmed the hepatic artery proximally and distally and blocked it with an artery clamp to prepare the anastomosis under a microscope (Figure 3). First, we anastomosed the distal hepatic artery, fully flushed the arterial lumen with heparin-containing saline, and intermittently anastomosed one artery end with 8-0 Prolene, suturing at 0° and 180°, respectively. For traction, we added a needle between the first two sutures [2]. We flipped the artery after finishing the anterior wall anastomosis and then anastomosed the posterior wall in the same manner. We used the same method to anastomose the other end of the hepatic artery and the artery bridge. During the anastomosis, we ensured that each needle is inserted perpendicularly to penetrate the whole arterial layer to avoid bringing any artery adventitia into the lumen. After completing the anastomosis, we opened the artery clips to check for blood leakage (Figure 4). After surgery, we used conventional low molecular weight heparin (5000 U/day) as an intravenous continuous infusion anticoagulant to prevent the formation of hepatic artery thrombosis. Routine bedside B ultrasound was performed once daily to observe the arterial flow. We also monitored the blood coagulation and blood chemistry testing results. One week after surgery, we reexamined the patient by enhanced abdominal CT. Postoperative follow-up is performed thereafter. The specific surgical artery reconstruction methods are shown in Table 1.

## 3. Results

In the nine patients, the affected arteries were all right hepatic arteries, and artery bridges for hepatic artery reconstruction were performed using intermittent 8-0 Prolene for the anastomosis. There were no abnormalities in the postoperative stomach tube drainage or gastrointestinal motility. Routine enhanced abdominal CT was performed at one week, one month, and three months after surgery. No complications, such as arterial thrombosis, bile leakage, and liver dysfunction, were observed during the long-term postoperative follow-up.

For all the nine patients, the arterial reconstructions were intermittently anastomosed using a surgical loupe (≥2.5x) with 8-0 Prolene. During the anastomosis, the assistant repeatedly flushed the arterial lumen with heparin saline to maintain a clear lumen. The affected lengths of the arteries in all the patients were ≥2 cm, so arterial graft bridging was needed to ensure an arterial tension-free anastomosis. All arterial reconstruction surgeries were anastomosed successfully at first attempt. The mean time of the anastomosis was 23 ± 3.1 min.

## 4. Discussion

Surgical resection is still the preferred treatment for hilar cholangiocarcinoma [3–5]. However, whether tumor radical resection can be achieved depends on the situation of affected tissues or organs by the tumor, such as blood vessels, nerves, and/or biliary ducts and if the patient has detectable liver and/or peritoneal metastases. The affected artery is generally determined by preoperative imaging and exploration during surgery. Preoperative imaging mainly utilizes enhanced CT, MRI, and angiography. Enhanced arterial phase images manifest as artery deformation due to extrusion, without a smooth arterial lumen, and a significantly reduced blood supply in the distal end of the tumor. During the intraoperative exploration, if the hepatic artery is surrounded by tumor or the arterial outer membrane is affected, a white arterial adventitia can be observed, indicating that the artery is affected by tumor and needs resection and reconstruction.

In cases of hilar cholangiocarcinoma, when the tumor affects the hilar vessels, the need for extended radical surgery, such as combined resection of the affected vessels, has gradually been recognized, since such procedures have been shown to help reduce the chance of tumor recurrence and improve the cure rate and long-term survival [6, 7]. Of note, some scholars believe that it is unnecessary to reconstruct the affected hepatic arteries after resection [8]. More recently, studies have shown that, in hilar cholangiocarcinoma surgery, patients undergoing combined tumor and artery resection, but without reconstruction, have a risk of developing liver necrosis and abscess after surgery [9], which is often fatal. In addition, the incidence of postoperative biliary complications among these patients undergoing hepatic artery reconstruction after resection is about 20%, while the same figure in patients without reconstruction after hepatic artery resection is almost 100% [10], indicating that ensuring an adequate hepatic arterial blood supply not only guarantees a better liver function, but also helps reduce postoperative biliary complications [11].

In this group of patients, the affected arteries were all right hepatic arteries, which is believed to be mainly due to the anatomical distribution of the hepatic artery that is mainly located behind the biliary ducts and vulnerable to being affected by biliary tumors. Our previous experience with surgical treatment of hilar cholangiocarcinoma showed that* in situ* arterial reconstruction can be chosen for an affected arterial length of ≤1 cm; that is, the remaining hepatic artery should be fully freed and then reconstructed* in situ*. This would ensure tension-free arterial reconstruction and avoid unnecessary surgical procedures. For the cases with an affected artery ranging between 1 and 2 cm, it is possible to flip the gastroduodenal artery upward after cutting the distal end and then anastomosing it to the remaining hepatic artery. In this case, it is sometimes necessary to cut off the gastroduodenal artery to indirectly extend the length of the remaining artery in order to ensure that a tension-free anastomosis can be made. In cases with an affected hepatic artery length of ≥2 cm, the above method cannot guarantee a tension-free anastomosis, and, therefore, it is necessary to perform arterial bridge reconstruction.

In the previous reports of hilar cholangiocarcinoma surgery with hepatic artery resection, the vascular graft bridge for hepatic artery reconstruction generally used vein grafts or ectopic grafts [12–14]. Our results demonstrated the following points with regard to the bridging hepatic artery reconstruction: first, the nearest parts and* in situ* reconstruction were employed to fully guarantee* in situ* liver hemodynamic needs; second, the procedure was simple and convenient, with no gastrointestinal or other complications observed in our patients; third, the gastroduodenal artery and hepatic artery diameters matched well, making it easy to conduct the anastomosis; and, fourth, the procedure avoided surgical procedures involving ectopic parts, preventing cross-infection, which is also recognized to be associated with the tumor metastasis [15]. Therefore, to ensure radical resection of these tumors, the resection of the affected hepatic artery, combined with hepatic artery* in situ* reconstruction, is a key factor for improving the surgical outcome and quality of life of the patient. Our results indicated that selecting a gastroduodenal artery graft as a bridge is a good choice for hepatic artery reconstruction in patients undergoing surgery for hilar cholangiocarcinoma.

## Figures and Tables

**Table 1:** Gastroduodenal artery bridging for hepatic artery reconstruction.

Hilar cholangiocarcinoma type	Number of cases	Surgical methods	Affected artery length (cm)
Bismuth IIIa	2	Liver quadrate lobe + caudate lobe resection	2, 2.5
Bismuth IIIb	6	Left liver + caudate lobe resection	2, 2.5, 2, 2.3, 2, 2.1
Bismuth IV	1	Liver quadrate lobe + caudate lobe resection	2.5
